# Combe on the Influence of Sleep in Warding off Insanity

**Published:** 1844-11

**Authors:** 


*Combe on the Influence of Sleep in warding off Insanity.*—"If those who are exposed to any of the exciting causes of cerebral disease, or of insanity, put themselves on their guard to secure regular sound sleep, they will do much to ward off an attack. The moment the cause begins to excite sleeplessness by night, and restlessness by day, with an involuntary propension of the mind in one direction, at first perceptible perhaps only to the patient himself, it is time to take alarm, and if possible, remove or counteract its agency. If it is excessive application to business, continued anxiety of mind, or excess of study, that is keeping up the activity of the brain, and placing it on the verge of disease, this may often be prevented by timely relaxation, or removal from the scene of anxiety, and particularly by carrying off much of the nervous energy in abundant muscular exercise often repeated, and by rigidly abstaining from mental exertion at night, and thereby allowing the brain to fall into that state of quiescence most favorable for repose. I have seen some striking instances of the efficacy of this plan in restoring tranquillity of mind, when on the very verge of derangement. The excitement of company and of tea, sometime resorted to in such instances, may, if carried to any length, only add fresh fuel to the flame, and stimulate the brain beyond recovery; but the society of those whose feelings and pursuits are calculated to soothe those most excited in the patient, and to call others into action, is very beneficial."—*Ibid.*

